# Barnidipine Real-Life Efficacy and Tolerability in Arterial Hypertension: Results from Younger and Older Patients in the BASIC-HT Study

**DOI:** 10.2174/1874192401711010120

**Published:** 2017-11-17

**Authors:** Robert Lins, Caroline De Vries

**Affiliations:** 1 Department of Internal Medicine, University of Antwerp, Antwerp, Belgium; 2 Astellas Pharma B.V., Leiden, The Netherlands

**Keywords:** Calcium channel blockers, Barnidipine, Age, Hypertension, Efficacy, Tolerability

## Abstract

**Objective:**

The aim of this study was to compare the efficacy and tolerability of barnidipine, a strong lipophilic calcium channel blocker, in younger (≤55 for efficacy or <65 years for adverse events) versus older (>55 or ≥65 years) patients with uncomplicated hypertension.

**Methods:**

20,275 patients received barnidipine, 10 or 20 mg/day, as monotherapy or in combination with other antihypertensive drug(s) in the observational BArnidipine real-life Safety and tolerability In Chronic HyperTension (BASIC-HT) study. Efficacy and tolerability were assessed over a 3-month period. The present paper describes results from prespecified subgroup analyses by age not reported elsewhere.

**Results:**

Both age groups showed a clinically meaningful decrease in blood pressure (BP) over time (p<0.0001). The mean systolic and diastolic BP after approximately 3 months of barnidipine therapy was well below the target value of <140/90 mmHg for individual patients, with no notable differences between age groups. The decrease in mean pulse pressure was greater in patients >55 years (-10.8 mmHg) than in patients ≤55 years (-8.7 mmHg) (p<0.0001) and the proportion of patients with pulse pressure >60 mmHg decreased from 61.1% at baseline to 24.8% at Visit 3 in patients >55 years and from 47.7% to 16.5% in patients ≤55 years (p<0.0001).

The overall incidence of adverse events was low, leading to treatment discontinuation in only 3.0-3.6% of patients. Peripheral edema, a common adverse effect with calcium channel blockers in clinical practice, was reported by 2.7% of patients aged <65 years and by 4.6% of patients aged ≥65 years.

**Conclusion:**

The efficacy and tolerability profiles of barnidipine as monotherapy or in combination with other antihypertensive drugs were shown to be favorable in both younger and older patients in a real-life practice setting. Randomized double-blind controlled studies are needed to confirm these results.

## 1 INTRODUCTION

Hypertension has become increasingly prevalent in recent years due to increased longevity and other contributing life-style factors such as obesity, physical inactivity and an unhealthy diet [1-3]. Hypertension is a major risk factor for cardiovascular disease and it is well established that control of blood pressure (BP) reduces the risk of myocardial infarction and stroke [4, 5]. The 2013 joint guidelines of the European Society for Hypertension/European Society for Cardiology (ESH/ESC) suggest, along with a comprehensive approach to cardiovascular risk management, a target BP of <140/90 mmHg for patients with hypertension [6]. BP control is however often poorly achieved in clinical practice [7-9]. Although monotherapy may sometimes suffice, mostly a combination of antihypertensive drugs from different classes is required to obtain good control [6, 10, 11].

According to the 2011 British Hypertension Society/National Institute of Clinical Excellence (NICE) guidelines for the clinical management of primary hypertension in adults [10], monotherapy with calcium channel blockers is indicated as a first-line treatment for patients aged 55 years or older with uncomplicated hypertension, unless such treatment is not suitable, for example because of edema or intolerance, or if there is evidence of heart failure or a high risk of heart failure [6, 11]. Calcium channel blockers either alone or in combination with other agent classes are also recommended by the 2013 joint guidelines of the ESH/ESC in low-to-moderate risk patients with uncomplicated hypertension [6] and are among the first choice options in the guidelines of the ESH/ESC and the U.S. eighth Joint National Committee (JNC 8) [6, 11].

Barnidipine is a long-acting and strong lipophilic dihydropyridine calcium channel blocker, with similar clinical efficacy to amlodipine and other calcium channel blockers [12-15] and is effective in combination with enalapril and losartan [16, 17]. Barnidipine has shown to be an effective antihypertensive agent in the management of hypertension in elderly patients [13, 18] and its efficacy is not negatively affected by age [19]. The tolerability of barnidipine is considered to be generally good or excellent in most patients, including the elderly (≥75 years) [13, 18, 19]. Barnidipine has one of the lowest discontinuation rates in its drug class [21]. The incidence of peripheral edema – one of the most common adverse events with calcium channel blockers especially in the elderly [22, 23] – is lower with barnidipine than with previous generation dihydropyridines [13, 20]. Other reported adverse events such as headache, flushing, vertigo and palpitations are mostly mild or moderate and transient in nature [13, 20] and are expected from its pharmacologic profile.

The tolerability and efficacy of barnidipine (10 or 20 mg) was studied in a large-scale open-label, prospective observational study in a population with essential hypertension, including patients with diabetic mellitus (n=20,279) (BASIC-HT; BArnidipine real-life Safety and tolerability In Chronic HyperTension) [24]. The results confirmed the efficacy of barnidipine in the real-life setting of routine clinical practice as an effective treatment for BP reduction. The results also showed the good tolerability profile of barnidipine.

The overall results from the BASIC-HT study were reported in 2015 [24]. The present paper describes the efficacy and tolerability of barnidipine monotherapy or combination treatment in elderly versus younger patients. These subgroup analyses by age were prespecified in the protocol and have not been reported elsewhere. Results were broken down in the age groups of patients aged ≤55 years and >55 years, as the age cut-off is of specific importance for BP target and for drug choice as a first-line treatment as per international guidelines [10, 11]. Frequencies of adverse events were broken down by the age groups of patients aged <65 years and ≥65 years, as patients with a chronological age of 65 years old or older are more likely to develop adverse events that are common with many calcium channel blockers, including edema and cardiac adverse events, such as tachycardia and/or palpitations [23, 25, 26].

## 2 MATERIALS AND METHODS

### 2.1 Design

The BASIC-HT study was a post-marketing open-label, prospective, observational study conducted in routine practice in a large population in Belgium and Luxembourg. The full methodology of BASIC-HT was previously described [24]. Eligible patients were those diagnosed with essential hypertension, including patients with diabetic mellitus, for whom barnidipine was considered to be a clinically appropriate treatment by the treating physician. The protocol was approved by an independent ethics committee [Clinical Research Ethical Committee (CREC) in Kortenberg, Belgium] and followed the Belgian code of deontology. Written informed consent was obtained from all patients before any study procedure was conducted.

Patients received barnidipine as monotherapy or in combination with other antihypertensive drug(s). Patients were prescribed barnidipine at the recommended dose of 10 mg orally once daily. If deemed necessary by the treating physician, a higher dose, i.e. 20 mg orally once daily, could be prescribed.

Efficacy was assessed from systolic BP (SBP) and diastolic BP (DBP) office recordings at baseline and at 2 additional routine visits, which took place during the 3-month follow-up period. At each visit, BP was measured on the same arm with a sphygmomanometer having a precision of 2 mmHg. Each measurement was repeated 3 times and the mean value of these measurements was used in the analyses. Pulse pressure was calculated as the difference between SBP and DBP.

Tolerability was assessed from the frequency and severity of adverse events and by recording of heart rates (HR). HR was recorded at the beginning and at the end of each visit and was measured in beats/min (bpm).

At each visit, the treating physician checked if any adverse event had occurred since the last visit. For each adverse event, a structured questionnaire was completed by the treating physician with a separate questionnaire for serious adverse events. Dropout rates were used to indirectly assess adherence to treatment. Treatment satisfaction was evaluated by the investigator based on the tolerability and efficacy after 3 months of therapy.

### 2.2 Statistical Analysis

The statistical analysis was performed using SAS software for WINDOWS version 9.2. Descriptive statistics of SBP, DBP, pulse pressure and HR at each visit were calculated for quantitative variables and consisted of the number of available and missing observations, mean, standard deviation (SD), minimum, maximum, median, and 95% confidence intervals (CIs). Frequency distributions were provided for ordinal and nominal variables and consisted of the numbers and percentages for each score or group.

BP and HR data were assessed in the subgroups of patients aged ≤55 years versus patients aged >55 years.

Adverse events were coded using the Medical Dictionary for Regulatory Activities (MedDRA) version 12.0. The frequencies of adverse events for younger (<65 years) and elderly patients (≥65 years) were compared using descriptive statistics.

## 3 RESULTS

### 3.1 Demographics and Other Baseline Characteristics

A total of 20,275 out of the 20,479 enrolled patients were included in the intent-to-treat (ITT) set, which comprised of all patients who were enrolled and treated, and for whom follow-up information was available. Subgroup analyses by age were performed on all patients from the ITT set for whom age was reported (n=19,558 in total; i.e., 10,313 patients <65 years and 9,245 patients ≥65 years).

Most patients were aged >55 years (n=14,023; 71.7%), with a mean age of 69.3 years (Table **1**). Approximately one-quarter of all patients (n=4,778; 24.4%) were aged between 55-64 years, and about half of the patients were aged ≥65 years (n=9,245; 47.3%) of which 3,597 patients (18.4%) were older than 75 years (data not shown).

The mean age of the patients aged ≤55 years (n=5,535; 28.3%) was 47.6 years. Although there were approximately equal numbers of males and females in the study, the younger age group comprised of more males (59%) than females (41%) while the older age group included more females (53%) than males (47%).

The mean body mass index (BMI) was comparable in both age groups. Older patients more often had diabetes than younger patients (18.7 *vs* 10.9%).

Barnidipine was more often prescribed as monotherapy in younger (63.4%) than in older patients (42.7%) (Table **2**). In contrast, barnidipine in combination with other antihypertensive drug(s) was prescribed more often in older (57.3%) than in younger patients (36.6%).

The start dose of barnidipine was 10 mg in the vast majority of patients, with no clinically relevant difference between age groups (n=5,132 patients ≤55 years; 93.3%; and n=12,772 patients >55 years; 91.7%). The remaining patients received a start dose of 20 mg (n=369; 6.7% and n=1,158; 8.3%, respectively). The percentages of patients in these dosage groups were calculated with the total number of patients for whom the age and also the dosage were reported (n=19,431 in total; n=5,501 younger and n=13,930 older patients).

The mean SBP and DBP values at baseline were 158.2/94.9 mmHg and 160.2/92.2 mmHg for the younger and older age group respectively, and were well above the target value of <140/90 mmHg [6], with no clinically meaningful differences between age groups (Table **3**).

The mean pulse pressure, however, was higher in patients >55 years compared with younger patients (68.1 and 63.3 mmHg, respectively; p<0.001), and more patients >55 years had a pulse pressure >60 mmHg at baseline (61.1% compared with 47.7% of patients aged ≤55 years).

The mean HR value at the end of the baseline visit in the 10 mg barnidipine dosage group was 75.5 bpm for patients aged ≤55 years and 74.2 for patients aged >55 years (95% confidence interval for group difference 0.98 < 1.27 <1.57; p<0.001).

### 3.2 Efficacy Analysis

There was a statistically significant and clinically meaningful decrease in mean SBP and mean DBP in both younger and older patients (aged >55 and ≤55 years, respectively) treated with barnidipine over the course of the 3-month follow-up period (p<0.0001), with no notable clinical differences between age groups (Table **3**). The mean SBP and DBP values at the end of the 3-month follow-up period were below the blood pressure target of <140/90 mmHg for individual patients.

**Fig. 1:**
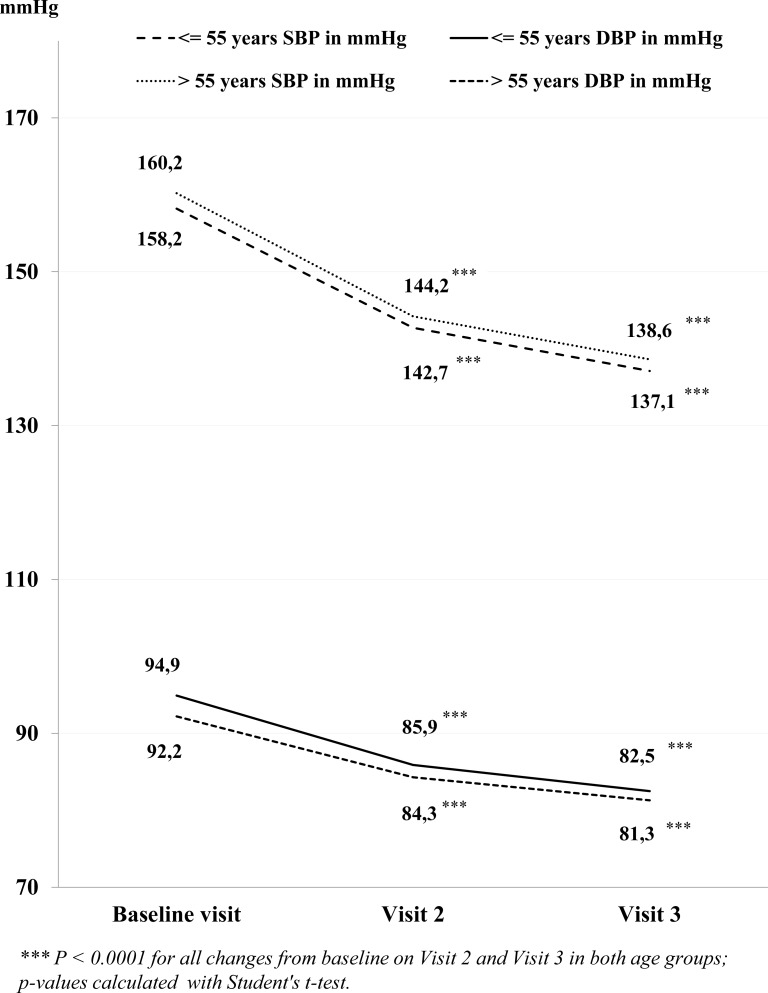
Mean systolic blood pressure (SBP) and diastolic blood pressure (DBP) at each visit for patients treated with barnidipine as monotherapy or in combination, split by age.

The mean change in SBP in older (>55 years) and younger (≤55 years) patients at Visit 2 was -16.0 mmHg and -15.5 mmHg, respectively, and the mean change in DBP was -7.9 mmHg and -9.0 mmHg, respectively (Fig. **1**).

A further decrease in SBP and DBP was observed at Visit 3, with a mean change from baseline of -21.6 mmHg and -21.1 mmHg, respectively for SBP, and a mean change from baseline of -10.9 mmHg and -12.4 mmHg, respectively, for DBP.

The decrease in mean pulse pressure after approximately 3 months of barnidipine therapy was larger in patients >55 years (-10.8 mmHg) compared with patients ≤55 years (-8.7 mmHg; p<0.0001). This trend was already visible at Visit 2 (-8.2 mmHg and -6.5 mmHg, respectively; p<0.0001).

### 3.3 Pulse Pressure

**Fig. 2:**
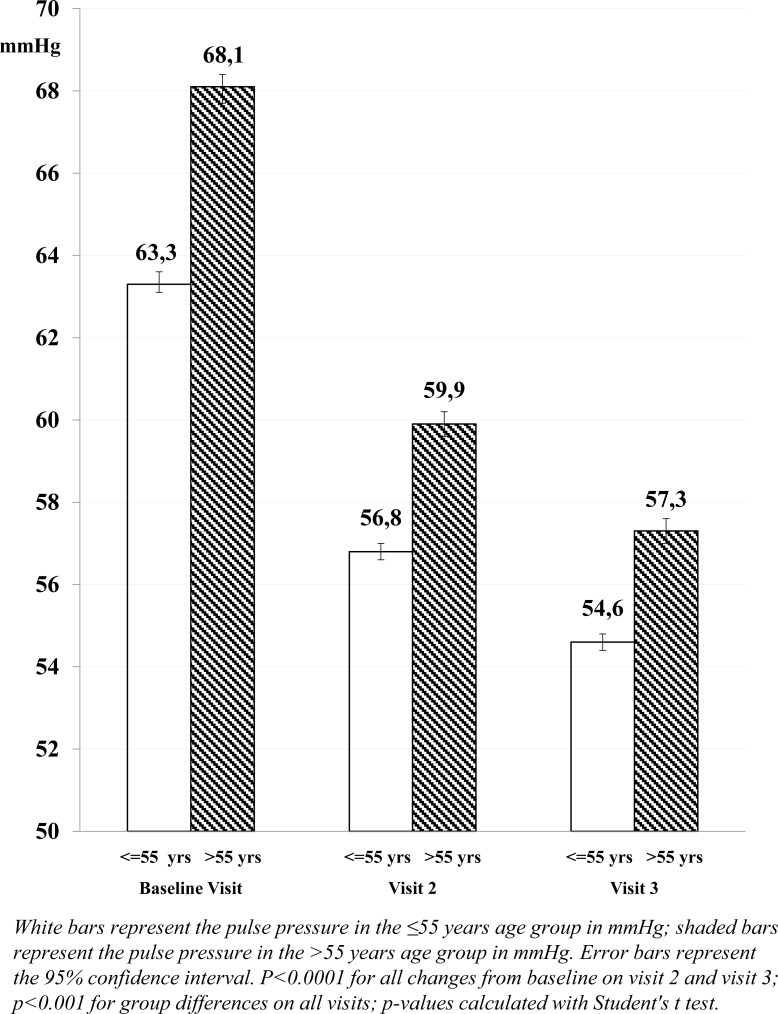
Mean pulse pressure at each visit for patients treated with barnidipine as monotherapy or in combination, split by age.

The proportion of patients with pulse pressure >60 mmHg decreased over the course of the 3-month follow-up period from 61.1% at baseline to 24.8% at Visit 3 in patients >55 years and from 47.7% to 16.5% in patients ≤55 years (Fig. **2**). The difference was considered clinically meaningful as elevated pulse pressure >60 mmHg is an established marker of adverse outcome in elderly patients with hypertension [27-29].

### 3.4 Heart Rate

The mean HR tended to decrease over the 3-month follow-up period for both older (>55 years) and younger patients (≤55 years) and for both dosage groups (10 mg and 20 mg). Overall, the decrease tended to be somewhat larger in younger patients than in older patients, and also somewhat larger with the 20 mg dosage than with the 10 mg dosage.

Younger patients who received a dose of 10 mg barnidipine had a change of -2.2 bpm (95% CI -2.0; -2.4) in HR whereas patients >55 years showed a mean change of -1.4 bpm (95% CI -1.30; -1.6). The difference was statistically significant (p<0.001).

Similarly, the mean reduction in HR in the age group of patients ≤55 years who received barnidipine 20 mg was -3.1 bpm (95% CI -2.3; -3.9) whereas the mean HR reduction in the older age group (>55 years) was -2.0 bpm (95% CI -1.5; -2,5, and p=0.074).

It should however be noted that baseline values in the age group of patients ≤55 years were higher than in patients >55 years, thereby leaving more room for improvement. Also, despite the smaller decrease from baseline, patients >55 years still had better (lower) mean HR values at the end of Visit 3 compared with patients ≤55 years.

### 3.5 Adverse Events

Barnidipine was well tolerated. The incidence of adverse events was low and generally comparable among age groups (Table **4**). Peripheral edema was the most commonly reported adverse event, and was observed in 2.7% of patients aged <65 years and in 4.6% of patients aged ≥65 years. Headache was reported in 1.8% of patients aged <65 years and in 1.4% of patients aged ≥65 years.

The incidence of vascular adverse events (flushing, hot flush) and cardiac adverse events (tachycardia, palpitations) was low (<1.0%) with no notable or clinically meaningful differences between age groups.

### 3.6 Dropout Rate

The vast majority of patients completed the 3-month follow-up period. The dropout rate was low, *i.e.* 447 (8.6%) patients aged ≤55 years and 989 (7.5%) patients aged >55 years, discontinued treatment at any time during the study. The dropout rates due to adverse events were 3.0% (168 out of 5,535) and 3.6% (501 of 18,642), respectively. Over 95% of patients intended to continue the barnidipine treatment after the end of the study.

### 3.7 Treatment Satisfaction

Efficacy was considered very good or good for approximately 93% of younger and older patients receiving barnidipine monotherapy and for approximately 90% of the patients receiving barnidipine in combination with other antihypertensive drugs (Table **5**).

Likewise, the tolerability of barnidipine was very good or good in over 92% of patients, with no relevant differences between age groups.

## 4 DISCUSSION

Hypertension is a major risk factor for the development of cardiovascular disease, and unlike other risk factors such as race or family history, can be controlled, treated or modified. As BP tends to rise with age, BP control in the elderly may significantly contribute to reduced mortality and morbidity in this age group [6, 10, 11].

Several current international hypertension guidelines recommend that age should affect drug choices in a stepwise approach [10, 11].

With advanced age, systolic hypertension becomes predominant, which is largely accounted for by loss of elasticity and increasing rigidity of large arteries. Also, in elderly patients, the activity of the renin-angiotensin system is generally suppressed, and as a consequence BP reduction with an angiotensin converting enzyme (ACE) inhibitor or an angiotensin II receptor blocker may be small [30, 31]. Therefore, calcium channel blockers are the preferred first-line treatment for patients aged 55 years or older as monotherapy (according to the NICE guidelines) [10] or among the first choice options in the guidelines of the ESH/ESC [6] and the JNC 8 [11].

If more than one drug is needed, combination therapy with a calcium channel blocker and either an ACE inhibitor or an angiotensin II receptor blocker is recommended. This combination has been proven superior to other combinations (e.g. a β-blocker plus a diuretic, or an ACE inhibitor/angiotensin II receptor blocker plus a diuretic) in patients of all ages, as it shows a lower incidence of cardiovascular events and adverse events, while it has similar effects in lowering BP and preserving renal function [32-34]. In keeping with these results, Falaschetti et al. reported that BP management and control between 1994 and 2011 in England improved when the most commonly prescribed combination therapies changed from diuretics plus β-blockers to renin-angiotensin system blockers plus diuretics in 2006, and to renin-angiotensin system blockers plus calcium channel blockers in 2011 [35]. Awareness, treatment, and control rates progressively improved across each stage of this 17-year period, with the prevalence of control across all treated patients almost doubling from 33% in 1994 to 63% in 2011. Nevertheless, of all adults in 2011, hypertension was controlled in only 37% [35]. Similar percentages (i.e. 22-42% for men, and 27-39% for women, depending on age) were recently reported for BP control in treated patients in high-income countries [9]. Therefore, hypertension awareness and appropriate treatment schemes, that avoid therapeutic inertia and enhance adherence, must continuously be the main focus.

Barnidipine is a strong lipophilic calcium channel blocker with efficacy similar to other dihydropyridines [12, 13]. The tolerability and efficacy of barnidipine (10 or 20 mg) given alone or in combination with one or more other antihypertensive drugs was recently studied in the real-life setting of routine clinical practice. Over 20,000 patients with hypertension were enrolled in the BASIC-HT study [24]. The overall results from this open-label prospective observational study showed that barnidipine is an effective treatment for BP reduction, comparable to the results obtained from randomized studies [13, 24]. The results also confirmed the good tolerability profile of barnidipine in a real life setting. The present paper describes for the first time the results from the subgroup analysis by age that was prespecified in the protocol and has not been reported elsewhere. Most patients (71.7%) in the BASIC-HT study were aged >55 years. Approximately one-quarter of all patients (24.4%) were aged between 55-64 years, about half of the patients were aged ≥65 years (47.3%), and 18.4% of patients were older than 75 years. The age cut-offs that were used for the subgroup analyses (≤55 *vs* >55 years and <65 *vs* ≥65 years) were based on several current international hypertension guidelines that have dichotomized age groups around a cut-off value of 55-60 years for choice of first-line treatment [6, 11] and on literature data that suggest that elderly patients (i.e. those with a chronological age of 65 years old or older) are more likely to develop adverse events that are common with many calcium channel blockers [23, 25, 26].

The subgroup analysis by age showed a statistically significant and clinically meaningful decrease in mean SBP and DBP in both younger and older patients over the course of the 3-month follow-up period. The mean SBP and DBP values at the end of the 3-month follow-up period were below the BP target of <140/90 mmHg for individual patients, i.e. 137.1/82.5 mmHg for patients ≤55 years, and 138.6/81.3 mmHg for patients >55 years. The results of the SPRINT study [36] showed that a further reduction (i.e. a lower SBP target of <120 mmHg; in an unattended automated office BP measurement protocol, so rather compared with <135 mmHg SBP [37]) reduced the rates of fatal and nonfatal major cardiovascular events and death from any cause in a patient population of ≥50 years to a larger extent compared to a SBP target of <140 mmHg [36]. However, in the SPRINT study, a more intensive BP management regimen was associated with higher rates of serious adverse events of hypotension, syncope, electrolyte abnormalities, and acute kidney injury or failure [36]. Such an intensified approach may therefore be not safe enough for treatment of elderly, more fragile patients with co-morbidities. In a prespecified subgroup of elderly aged 75 years or older in the SPRINT study, the beneficial results of lower BP targets were confirmed, but the frail elderly remain a risk group [38].

In older patients with hypertension, pulse pressure appears to be a major determinant of cardiovascular risk related to arterial stiffness. Elevated pulse pressure (above 60 mmHg) in the elderly is a risk factor for asymptomatic organ damage [27-29]. In the present BASIC-HT study, a reduction in pulse pressure was observed after 1 and 3 months of barnidipine treatment. The reduction from baseline was larger in patients older than 55 years (-10.8 mmHg) compared with younger patients (-8.7 mmHg). Moreover, after approximately 3 months of treatment, only 24.8% of the older patients still presented with a pulse pressure of >60 mmHg, compared with 61.1% of patients at baseline. This is in line with an earlier study with barnidipine, in which the pulse pressure was significantly lowered to a mean of 58 mmHg after 6 months of treatment [39]_._

Various studies have reported a negative association between elevated resting HR and cardiovascular outcomes in hypertension, coronary artery disease and heart failure [40-42]. The mean resting HR in the present BASIC-HT study tended to decrease slightly with treatment duration for both older and younger patients. These results are in line with the results from earlier studies with barnidipine [38, 43] and supports the idea that barnidipine does not cause reflex neurohumoral activation [15].

Barnidipine was well tolerated. The incidence of adverse events was low and generally comparable among age groups, and less than 4% of patients dropped out due to adverse events. Events reported with the highest incidence (peripheral edema, headache, nausea, dizziness and flushing) are events generally reported in patients treated with calcium channel blockers and caused by drug-induced vasodilation.

Peripheral edema is one of the most common adverse effects with dihydropyridine calcium channel blockers in clinical practice and is likely to be related to the vasodilatory action of these drugs [22, 44, 45]. The incidence rates for peripheral edema reported in the literature range from 3-30%, partly also because the effect may be more common in women [23, 26, 45] and also varies (among others) with age [19, 25] and the type and dose of the calcium channel blocker [22, 23, 45-47]. While calcium channel blocker-associated edema is not life-threatening, it is distressing to many patients, and may contribute to an increased dropout rate and decreased compliance with therapy [45] or can even effectively deter a clinician from prescribing these drugs [26]. The incidence of peripheral edema with barnidipine in the BASIC-HT study was low (*i.e.* observed in only 4.6% of patients aged 65 years or older) and comparable with that observed in randomized clinical studies including elderly and very elderly hypertensive patients [19, 20]. It has been hypothesized that this low incidence may be due to the slow onset of action of barnidipine and the fact that it has higher affinity for smooth muscles in the mesenteric and renal tissue vascular beds than for peripheral arteries in the extremities [12, 48]. The low incidence as observed in the present BASIC-HT study may also partly be due to the fact that more than half (54.1%) of the patients older than 55 years of age in were prescribed barnidipine in combination with another antihypertensive therapy. If these were combinations with an ACE inhibitor or with an angiotensin receptor blocker, as recommended in current international hypertension guidelines, the incidence rates of calcium channel blocker-related adverse events would be typically reduced compared with monotherapy [22, 46, 49]. The other hypertensive drugs that were prescribed in combination therapy with barnidipine in the present BASIC-HT study were however not further specified, so no distinct conclusions can be drawn.

Cardiac side effects such as tachycardia and/or palpitations are common with dihydropyridine calcium channel blockers in general. In the present BASIC-HT study, the incidence of cardiac adverse events was very low (≤0.5%) with no notable or clinically meaningful differences between older and younger patients. This is in line with previous studies with barnidipine, and is expected from its pharmacologic profile [12, 13].

Efficacy and tolerability were considered by the investigator to be very good or good in over 92% of patients.

In summary, the results from the present analysis show that barnidipine monotherapy or when given in combination with antihypertensive drugs from other drug classes can safely and effectively be used for treatment of patients with uncomplicated mild to moderate hypertension, including the elderly population. The mean SBP and DBP values at the end of the 3-month follow-up period were below the BP target of <140/90 mmHg for individual patients. Barnidipine monotherapy or as part of combination therapy was well tolerated. The incidence of peripheral edema, one of the most common adverse effects with dihydropyridine calcium channel blockers and often the cause of treatment discontinuation, was low in both age groups. Based on the results of the present study, the risk-benefit profile of barnidipine is considered favorable in both younger and older patients. Additional randomized double-blind controlled studies are needed to confirm these results.

## CONCLUSION

The efficacy and tolerability profiles of barnidipine as monotherapy or in combination with other antihypertensive drugs in keeping with current international treatment guidelines were shown to be similar in younger and older patients with hypertension in a real-life practice setting. Barnidipine can therefore be effectively and safely used in patients of all ages, including elderly patients. The low incidences of peripheral edema, palpitations and tachycardia, suggest a role of barnidipine as antihypertensive drug for patients not tolerating other calcium channel blockers and patients at greater cardiovascular risk.

## Figures and Tables

**Table 1:** Demographic and other patient characteristics at baseline visit, split by age.

–	–	**≤55 years**		**>55 years**	
**Age (years)**	n (%)	5,535	(28.3%)	14,023	(71.7%)
	mean (SD)	47.6	(6.5)	69.3	(8.7)
**Weight (kg)**	n	5,441		13,799	
	mean (SD)	82.3	(15.7)	77.2	(13.7)
**Body mass index (kg/m^2^)**	n	5,418		13,684	
	mean (SD)	27.7	(4.8)	27.3	(4.4)
**Gender**	n	5,512		13,980	
**male**	n (%)	3,253	(59%)	6,524	(47%)
**female**	n (%)	2,259	(41%)	7,456	(53%)
**Diabetes status**	n	5,458		13,771	
**yes**	n (%)	596	(10.9%)	2,579	(18.7%)
**SD: Standard deviation**					

**Table 2:** Prescription of barnidipine treatment, split by age.

–	–	**≤55 years**	**>55 years**
**Monotherapy**	n (%)	2,322 (63.4%)	3,985 (42.7%)
**Combination with other antihypertensive drug(s)**	n (%)	1,341 (36.6%)	5,343 (57.3%)

**Table 3:** Systolic and diastolic blood pressure values and changes from baseline, split by age.

–	–	–	**≤55 years**	**>55 years**
**Systolic Blood**	Baseline	n	5,514	13,959
**Pressure (mmHg)**		mean (SD)	158.2 (13.9)	160.2 (14.9)
		95% CI	**157.8; 158.6**	**160.0; 160.5**
	Visit 2	n	5,486	13,893
		mean (SD)	142.7 (12.6)	144.2 (12.8)
		95% CI	**142.3; 143.0**	**144.0; 144.4**
		Change from baseline	-15.5 (p<0.0001)	-16.0 (p<0.0001)
	Visit 3	n	5,049	12,889
		mean (SD)	137.1 (11.3)	138.6 (11.3)
		95% CI	**136.8; 137.4**	**138.4; 138.8**
		Change from baseline	-21.1 (p<0.0001)	-21.6 (p<0.0001)
**Diastolic blood**	Baseline	n	5,514	13,959
**Pressure (mmHg)**		mean (SD)	94.9 (9.0)	92.2 (9.2)
		95% CI	**94.6; 95.1**	**92.0; 92.3**
	Visit 2	n	5,486	13,893
		mean (SD)	85.9 (8.2)	84.3 (7.9)
		95% CI	**85.6; 86.1**	**84.2; 84.4**
		Change from baseline	-9.0 (p<0.0001)	-7.9 (p<0.0001)
	Visit 3	n	5,049	12,889
		mean (SD)	82.5 (7.2)	81.3 (6.9)
		95% CI	**82.3; 82.7**	**81.2; 81.5**
		Change from baseline	-12.4 (p<0.0001)	-10.9 (p<0.0001)
**Pulse pressure**	Baseline	n	5,514	13,959
**(mmHg)**		mean (SD)	63.3 (13.8)	68.1 (14.9)
		95% CI	**63.0; 63.7**	**67.8; 68.3**
	Visit 2	n	5,486	13,893
		mean (SD)	56.8 (11.6)	59.9 (12.0)
		95% CI	**56.5; 57.1**	**59.7; 60.1**
		Change from baseline	-6.5 (p<0.0001)	-8.2 (p<0.0001)
	Visit 3	n	5,049	12,889
		mean (SD)	54.6 (10.3)	57.3 (10.9)
		95% CI	**54.3; 54.9**	**57.1; 57.5**
		Change from baseline	-8.7 (p<0.0001)	-10.8 (p<0.0001)
**95% CIs which do not overlap between groups are in bold; SD: Standard deviation; CI: confidence interval; ** **p-values for change from baseline calculated with Student's t-test**				

**Table 4:** Number and percentage of patients with adverse events (incidence ≥0.1%), split by age.

–	**<65 years (n=10,313)**	**≥65 years (n=9,245)**
**Adverse Event**	**Number of Patients (%)**	**Number of Patients (%)**
Edema peripheral	281 (2.7%)	426 (4.6%)
Headache	190 (1.8%)	130 (1.4%)
Flushing	94 (0.9%)	63 (0.7%)
Dizziness	64 (0.6%)	56 (0.6%)
Palpitations	50 (0.5%)	26 (0.3%)
Hot flush	43 (0.4%)	24 (0.3%)
Nausea	38 (0.4%)	52 (0.6%)
Erythema	30 (0.3%)	21 (0.2%)
Edema	21 (0.2%)	51 (0.6%)
Tachycardia	17 (0.2%)	21 (0.2%)
Fatigue	10 (0.1%)	11 (0.1%)
Malaise	6 (0.1%)	11 (0.1%)

**Table 5:** Investigator opinion of efficacy and tolerance on barnidipine at the end of the study visit (Visit 3).

–	**Monotherapy**				**Combination Therapy**			
–	≤55 years		>55 years		≤55 years		>55 years	
**Efficacy, n (%)**								
**Very Good**	1,238	(53.6%)	2,039	(51.5%)	583	(43.4%)	2,309	(43.4%)
**Good**	911	(39.5%)	1,678	(42.3%)	619	(46.1%)	2,508	(47.1%)
**Moderate**	135	(5.9%)	207	(5.2%)	125	(9.3%)	446	(8.4%)
**Not Good**	25	(1.1%)	39	(1.0%)	15	(1.1%)	59	(1.1%)
**Tolerance n (%)**								
**Very Good**	1,402	(62.1%)	2,353	(61.1%)	659	(54.2%)	2,672	(54.7%)
**Good**	747	(33.1%)	1,266	(32.9%)	478	(39.3%)	1,854	(37.9%)
**Moderate**	64	(2.8%)	139	(3.6%)	46	(3.8%)	232	(4.8%)
**Not Good**	43	(1.9%)	90	(2.3%)	33	(2.7%)	130	(2.7%)
